# A Novel Method of Improving the Mechanical Properties of Propellant Using Energetic Thermoplastic Elastomers with Bonding Groups

**DOI:** 10.3390/polym16060792

**Published:** 2024-03-13

**Authors:** Shixiong Sun, Haoyu Liu, Yang Wang, Wenhao Du, Benbo Zhao, Yunjun Luo

**Affiliations:** 1 School of Chemistry and Chemical Engineering, North University of China, Taiyuan 030051, China; sunshixiong1989@126.com (S.S.); liuhaoyu8091@163.com (H.L.); 15227110251@163.com (Y.W.); wenhao_du@outlook.com (W.D.); 2 Dezhou Industrial Technology Research Institute of North University of China, Dezhou 253034, China; 3 School of Materials Science and Technology, Beijing Institute of Technology, Beijing 100081, China

**Keywords:** EMDB propellant, GAP-ETPE, interface properties, mechanical performance

## Abstract

The relatively poor mechanical properties of extruded modified double base (EMDB) propellants limit their range of applications. To overcome these drawbacks, a novel method was proposed to introduce glycidyl azide polymer-based energetic thermoplastic elastomers (GAP-ETPE) with bonding groups into the propellant adhesive. The influence of the molecular structure of three kinds of elastomers on the mechanical properties of the resultant propellant was analyzed. It was found that the mechanical properties of the propellant with 3% CBA-ETPE (a type of GAP-ETPE that features chain extensions using N-(2-Cyanoethyl) diethanolamine and 1,4-butanediol) were improved at both 50 °C and −40 °C compared to a control propellant without GAP-ETPE. The elongation and impact strength of the propellant at −40 °C were 7.49% and 6.58 MPa, respectively, while the impact strength and maximum tensile strength of the propellant at 50 °C reached 21.1 MPa and 1.19 MPa, respectively. In addition, all three types of GAP-ETPE improved the safety of EMDB propellants. The friction sensitivity of the propellant with 3% CBA-ETPE was found to be 0%, and its characteristic drop height *H*_50_ was found to be 39.0 cm; 126% higher than the traditional EMDB propellant. These results provide guidance for studies aiming to optimize the performance of EMDB propellants.

## 1. Introduction

Extruded modified double-base (EMDB) propellants with aluminum (Al) and hexogen (RDX) particles are one of the commonly used solid propellants for tactical rockets due their numerous advantages, including low cost, high production efficiency, mature process, and consistency between batches [1]. However, these propellants usually have poor mechanical properties, especially with regard to their toughness at low temperatures. In addition, the elongation of the propellants is only about 3% and their impact strength is only about 3 MPa at −40 °C when the solid particle content of the propellant is as high as 55% [2,3]. These are primarily due to the structure of the adhesive of EMDB propellants. Nitrocellulose (NC) is a rigid polymer used as the adhesive skeleton of these propellants; this gives the material its low toughness due to the nitrocellulose/nitroglycerin (NC/NG) adhesive system [4,5]. However, the interface character between the double base adhesive and RDX is poor, resulting in low adhesion. This weak interface is another weakness in the EMDB propellant that further reduces its mechanical properties [1]. Furthermore, NG is very sensitive, making the preparation and use of the EMDB propellant more dangerous. These drawbacks limit the use of EMDB propellants in a variety of applications.

Two methods can be used to improve the mechanical properties of EMDB propellants. First, the use of NG can be substituted with other nitrate esters such as diethyleneglycol dinitrate (DEGDN), triethylenglycol dinitrate (TEGDN), trimethylolethane trinitrate (TMETN), Butyl Nitroxyethyl Nitramine (Bu-NENA) [4,6,7]. Indeed, some nitrate esters with lower sensitivity can plasticize NC better than NG. This would not only improve the mechanical properties of the propellant but also reduce its sensitivity. This is a common strategy in the regulation of the mechanical properties of propellants. However, those nitrate esters tend to reduce the high-temperature strength of EMDB propellants. A second method of improving the interface performance between NC/NG adhesive and RDX is the surface modification [8,9,10] of RDX with techniques such as spheroidization and surface coating. Although this process generally improves the mechanical properties of the target propellants, it is relatively complex. We previously introduced Bu-NENA, an insensitive nitrate ester with improved flexibility, into an EMDB propellant named R3 [11]. The elongation of the R3 propellant at −40 °C increased by a factor of 2 from 3.54% to 7.09%. In addition, the friction sensitivity of R3 dropped from 46% to 0%, while its *H*_50_ increased by 87.2% from 17.2 cm to 32.2 cm. In summary, the low-temperature mechanical properties and sensitivity of the EMDB propellant were improved significantly. Further improvements in mechanical properties could be achieved if the interface performance between the adhesive and RDX could be optimized.

Glycidyl azide polymer-based energetic thermoplastic elastomer (GAP-ETPE) is a linear polymer; propellants based on this polymer are known as green solid propellants due to the so-called 3R characteristics (recycle, recover, reuse) [12,13]. In our previous study, a series of GAP-ETPE with binding groups were synthesized [14]. Some of these materials were used as propellant adhesives when exploring the use of green or high-strength propellants [15]. An analysis of the phase structure revealed that NC molecules could diffuse into GAP-ETPE molecules due to the plasticization of Bu-NENA. Consequently, this suggests that GAP-ETPE with bonding groups could be dispersed into NC/Bu-NENA adhesives at the molecular level, improving its interface compatibility; this would result in improved mechanical properties in EMDB propellants with high-solid contents.

This study proposes a novel method of toughening the EMDB propellant based on the advantages of GAP-ETPE with bonding groups. The influence of GAP-ETPE on the tensile and impact properties of the EMDB propellant was studied in the context of its molecular structure. It was found that the mechanical properties of the E3C propellant (with 3% CBA-ETPE) could be improved at 50 °C and −40 °C simultaneously. These results may provide insights into the improved performance of the EMDB propellant and the structural design of adhesives for a high-performance solid propellant.

Table 1 provides a list of abbreviations used in this manuscript.

## 2. Materials and Methods

### 2.1. Experimental Materials

**Figure 1:**
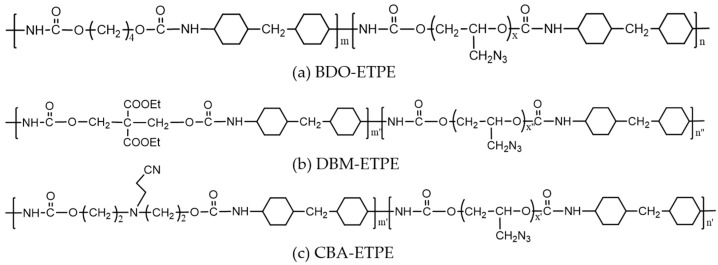
The molecular structure diagram of three kinds of GAP-ETPE: (**a**) BDO-ETPE; (**b**) DBM-ETPE; (**c**) CBA-ETPE.

Three kinds of GAP-ETPE with 30% hard-segment content were synthesized in the laboratory. Here, BDO-ETPE refers to an elastomer with BDO as a chain extender, while CBA-ETPE refers to an elastomer with its chain extended by BDO and CBA in a mass ratio of 1:1. DBM-ETPE refers to an elastomer with its chain extended by BDO and DBM in a mass ratio of 3:1. Molecular structure diagrams of these three types of elastomers are presented in Figure 1. The synthesis routes of these three types of GAP-ETPE are presented in Figure S1. The basic physical properties of these elastomers are shown in Table 2.

The NC used in this study had an N content of 12.0% and an average molecular weight of about 80,000; it was obtained from the Shanxi Xingan Chemical Plant, Taiyuan City, China. Bu-NENA is a light-yellow oily liquid with a melting point of −28.0 °C, a density of 1.21 g/cm^3^, and a heat of formation of 249 kJ/mol; it was obtained from the Liming Chemical Institute, Luoyang City, China. A 72-μm hexogen (RDX) was obtained from Gansu Silver Light Chemical Industry Group Co., Ltd., Baiyin City, China. A 3-μm spherical Al was obtained from Changyuan Mingyu Aluminium Industry Co., Ltd., Xinxiang City, China.

### 2.2. Sample Preparation

EMDB propellants with GAP-ETPE incorporated into their adhesive were prepared using a traditional solvent-free method. An EMDB propellant without GAP-ETPE (R3) was also prepared as a control. The detailed chemical composition of the prepared propellants is shown in Table 3. It should be noted that the R3 propellant was the previously optimized formula on which the series of three EMDB propellants with GAP-ETPE (ExB, ExD, and ExC) were prepared. The NC/Bu-NENA adhesive decreased with increasing GAP-ETPE content. The mass ratio of NC/Bu-NENA was held constant at 43/57, based on the results of previous experiments [11].

### 2.3. Experimental Instruments and Test Conditions

#### 2.3.1. Interface Property Test

The interface characteristics of the adhesives were measured with an OCA contact angle analyzer (Dataphysics Co., Stuttgart, Germany). The testing solvents included *N*,*N*-dimethylformamide (DMF), ethylene glycol, and diiodomethane.

#### 2.3.2. Tensile Property Test

The EMDB propellants were cut into dumbbell-shaped test specimens. The tensile properties of the propellants were measured using the AGS-J Electronic Universal Testing Machine (Shimadzu Corporation, Kyoto, Japan) according to the China Military Standard GJB 770B-2005 413.1 [16]. The test conditions were as follows: temperature −40 °C, 20 °C, and 50 °C at a tensile rate of 10 mm/min. The testing machine was equipped with a high–low-temperature test box through which the ambient temperature could be regulated.

#### 2.3.3. Impact Property Test

The impact resistance of the propellant samples was measured using a TCJ liquid crystal pendulum impact-testing machine from Jilin Taihe Testing Machine Co., Ltd. (Changchun, China). The test temperatures were −40 °C, 20 °C, and 50 °C; the pendulum energy was 4 J; the size of the sample was 4 mm × 6 mm × 60 mm, and the support span was 40 mm.

#### 2.3.4. Sensitivity Measurements

The friction sensitivity of the propellants was measured according to China Military Standard GJB 770B-2005 601.2 [16] using a pendulum friction apparatus (Beijing nachen Technology Co., Ltd., Beijing, China). The conditions were as follows: a pendulum weight of 1.5 kg; a swaying angle of 66°; a pressure of 2.45 MPa; and a sample mass of 20 ± 1 mg. The impact sensitivity was measured according to China Military Standard GJB 770B-2005 602.1 [16] using a drop-hammer apparatus (Beijing nachen Technology Co., Ltd., Beijing, China) using an up-and-down method. The conditions were as follows: a sample mass of 30 mg and a hammer weight of 2 kg.

## 3. Results

### 3.1. Interface Characteristics of the NC/NENA/ETPE Adhesive

Solid propellant is a composite material comprising adhesive as the continuous phase and solid filler as the dispersed phase. Its mechanical properties are related not only to the mechanical properties of the adhesive itself but also to the interaction between the adhesive and solid filler [17,18]. The characteristics of the interface between the adhesive and solid filler are an important factor that affects the mechanical properties of the propellant. The surface energy of the adhesive is most commonly obtained using the contact angle method based on Young’s equation [19,20,21]. In this study, the adhesion work between the adhesive and RDX was calculated using this method.

The adhesive systems were prepared as thin films. Diiodomethane, N,N-dimethylformamide, and ethylene glycol were used as reference liquids to test the contact angles of the four adhesive films at room temperature. The results of these tests are presented in Table 4. The interfacial tension and adhesion work between the Bu-NENA/NC/GAP-ETPE adhesive system and RDX were calculated [19,20,21] as shown in Table 5. A detailed explanation of the calculation process is presented in the supplementary file. Ad-R3 refers to the adhesive used in the R3 propellant.

Table 5 shows that the magnitude of the adhesion work between the adhesive and RDX is, in ascending order, as follows: Ad-E3B, Ad-E3D, then Ad-E3C. This was consistent with the order of adhesion work observed between the three types of GAP-ETPE and RDX (BDO-ETPE/RDX: 62 mJ·m^−2^; DBM- ETPE/RDX: 73 mJ·m^−2^; CBA-ETPE/RDX: 76 mJ·m^−2^). Table 5 also shows that the adhesion work between the Ad-E3B adhesive system and RDX is lower than that between the Ad-R3 adhesive system and RDX. However, after introducing GAP-ETPE with bonding groups, the adhesion work between the resultant adhesive system (Ad-E3C and Ad-E3D) and RDX is higher than that between Ad-R3 and RDX. These results indicate that the bonding groups can improve the interface performance between the adhesive of a modified double-base propellant and RDX. This improvement may be due to two reasons. First, these elastomers may have formed a homogeneous structure with the double-base adhesive system under the plasticizing effect of Bu-NENA [11]. The elastomers can then be dispersed at the molecular level, allowing them to form effective contact surfaces with RDX. Alternatively, elastomers with bonding groups may have formed strong intermolecular forces with RDX [14]. Specifically, induced force could be produced between the ester groups in DBM and the nitro groups in RDX. Similarly, there could also be an induced force between the cyanide group in CBA and the nitro groups in RDX. It is worth noting that the induced force is stronger in the latter bond due to the stronger polarity of the cyanide group [22] and the increased availability of interaction sites in the CBA-ETPE molecule. Consequently, CBA-ETPE with cyanide as the bonding group was found to be the strongest adhesive system for RDX.

### 3.2. Tensile Properties of Propellants with Different ETPE Contents

**Figure 2:**
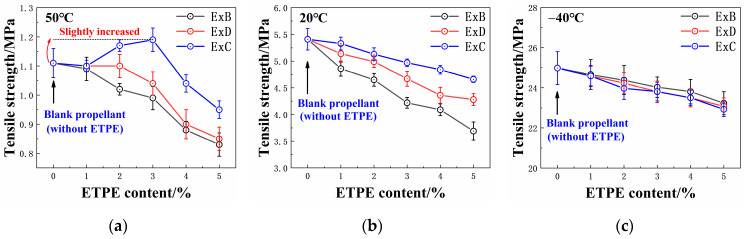
Tensile strength of the EMDB propellant with GAP-ETPE: (**a**) 50 °C; (**b**) 20 °C; (**c**) −40 °C.

**Figure 3:**
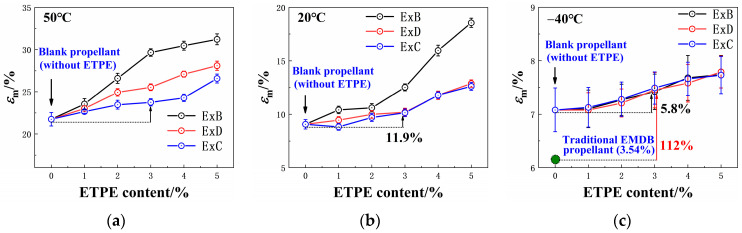
Elongation at maximum strength of EMDB propellant with GAP-ETPE: (**a**) 50 °C; (**b**) 20 °C; (**c**) −40 °C.

The tensile strength and elongation at maximum strength of the propellant are shown in Figure 2 and Figure 3, respectively. Detailed data on the tensile properties of each propellant can be found in Tables S1–S3. The *x* in “ExB” refers to the GAP-ETPE content in the EMDB propellant.

**Figure 4:**
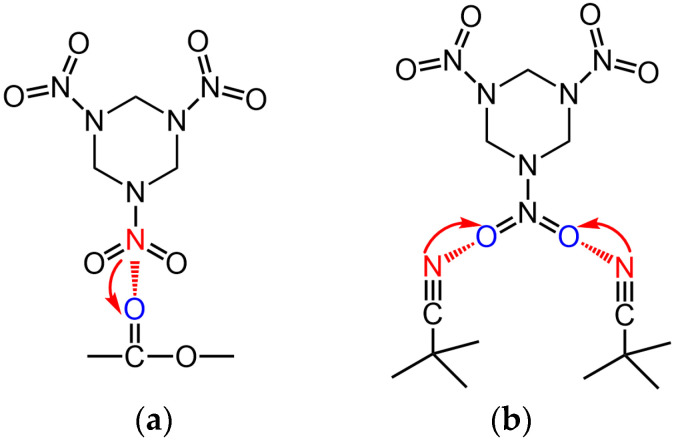
A schematic diagram of the induced forces between RDX and GAP-ETPE: (**a**) the induced force between RDX and DBM-ETPE; (**b**) the induced force between RDX and CBA-ETPE.

Figure 3 and Figure 4 show that the tensile strength of the ExB and ExD propellants at 50 °C decreased with increasing ETPE content, while ExC propellants exhibited an initial increase followed by a decreasing trend. The elongation at maximum strength gradually increased in all three propellants as the GAP-ETPE content increased.

Specifically, as the BDO-ETPE content increased, the tensile strength of the ExB propellant at 50 °C decreased while its elongation increased. This is because BDO-ETPE is a typical thermoplastic elastomer with a lower modulus and a greater toughness compared to the adhesive of double-base propellants [23]. The original plasticizing system changes as BDO-ETPE is partially substituted for Bu-NENA/NC in the binder system. Mutual diffusion occurs between NC and ETPE molecules under the plasticizing effect of Bu-NENA [24]. Consequently, a new homogeneous structure is formed comprising three components. Some hydrogen bonds and van der Waals forces between NC molecules are also disrupted by ETPE molecules. With increasing BDO-ETPE content, the reduction in hydrogen bonding within the original binder system is enhanced, leading to promoted molecular mobility [25,26]. Finally, the amount of NC also decreases. Consequently, the modulus of the adhesive declines, supported by the behavior of the tensile modulus shown in Tables S1–S3. In summary, the enrichment of GAP-ETPE in the propellant results in decreased tensile strength and increased elongation.

The trends observed In the ExD series of propellants with Increasing ETPE content were similar to those of the ExB series propellants. However, the tensile strength of ExD propellants was higher while their elongation was lower due to the COOEt group in the DBM molecule, which can induce interactions with the nitro group in RDX (as shown in Figure 4), enhancing the adhesion work between DBM-ETPE and RDX. As indicated in Table 3, the adhesion work between the binder and RDX in the E3D propellant is 72.59 mJ·m^−2^ compared to only 66.79 mJ·m^−2^ in the E3B propellant. This is clear evidence of the greater tensile strength of the ExD propellant compared to the ExB propellant.

As the CBA-ETPE content increases, the tensile strength of the ExC propellant initially increases before decreasing, while the elongation gradually increases. This behavior is due to the CN group in the CBA molecule, which can induce interactions with RDX (as shown in Figure 4). When CN groups were introduced into the binder system, the interactive forces at the binder–RDX interface were enhanced, leading to an increase in the tensile strength of the propellant [25,26]. However, the interfacial area between the adhesive and RDX and the content of the bonding groups in the elastomer was held constant throughout this process; consequently, the improvement in the mechanical properties of the propellant due to CBA-ETPE was non-linear. In addition, as the content of the elastomer increased, the tensile modulus of the binder system decreased (Tables S1–S3), resulting In the reduction of the mechanical properties of the propellant after the CBA-ETPE content exceeded 3% [1]. Since the adhesion work between CBA-ETPE and RDX is greater than that of DBM-ETPE and BDO-ETPE with RDX, the corresponding adhesion work between the Ad-ExC binder and RDX must also be greater than that of the latter two materials with RDX. Therefore, the ExC propellant has the highest tensile strength and lowest elongation compared to ExB and ExD propellants. It is important to consider *ε*_b_/*ε*_m_, known as the dewetting ratio [1]; a smaller dewetting ratio indicates better interface adhesion, as shown in Tables S1–S3. The ExC propellant has the smallest dewetting ratio, providing further evidence that this category of propellants exhibits the most desirable bonding effect with RDX for CBA-ETPE.

Figure 3 and Figure 4 show that as the temperature decreases, the structural differences of ETPE have less impact on the mechanical properties of the propellant. This is because the detachment of the binder and solid filler occurs prior to the internal fracture of the binder when the propellant is under external load [1,25]. These bonding groups, thus, increase the adhesive work between the binder and RDX, causing the propellant to preferentially undergo an internal tearing of the binder rather than detachment when subjected to external load. The binder system has a larger chain segment movement space at a relatively high temperature, making it prone to slipping between segments, resulting in larger deformations and easier interface detachment with RDX [26,27]. The bonding groups could enhance the interaction between the binder and RDX, preventing detachment in these scenarios. Consequently, these bonding groups have a significant impact on the mechanical properties of the propellant. However, the chain segment movement of the binder is restricted at low temperatures due to the significant intermolecular friction [26,27], making it more susceptible to chain fracture under external load. The weak interface between adhesive and RDX is not the key factor influencing the fracture of the propellant. In other words, the low-temperature tensile performance of the propellant was mainly affected by the content of ETPE rather than the type of ETPE.

### 3.3. Impact Mechanical Properties

**Figure 5:**
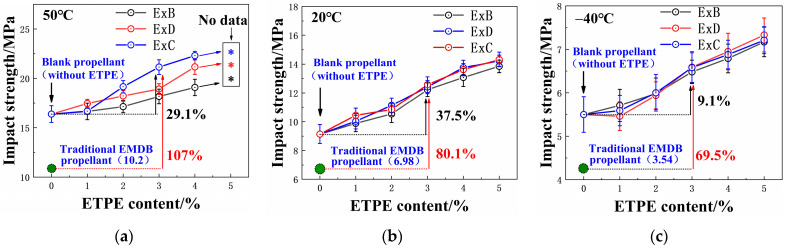
Impact strength of the EMDB propellant with GAP-ETPE: (**a**) 50 °C; (**b**) 20 °C; (**c**) −40 °C. No data refers to samples that were not broken into two pieces.

Figure 5 shows the impact strength of EMDB propellants with GAP-ETPE. Detailed data on the impact strength of the propellants can be found in Table S4. Figure 5 also shows that the impact strength of the three types of EMDB propellants gradually increased as the GAP-ETPE increased across all test temperatures. This may be primarily due to the influence of the characteristics of the molecular structure of the GAP-ETPE on the adhesive of the propellant. In the context of molecular structures, GAP-ETPE consists of two parts: a hard and soft segment. The soft segments mainly come from the polyether segments in GAP, which account for 70% of the total segments in the material. The hard segment is mainly composed of amino acid esters obtained by the reaction of isocyanates with chain extenders, accounting for 30% of the total material. The polyether segments are relatively flexible and exhibit good compatibility with NC. When introduced into the EMDB propellant, this material forms a blended system with NC under the plasticizing effect of Bu-NENA [11,28]. In this way, some hydrogen bonding and van der Waals forces between NC molecules are replaced by the van der Waals forces between ETPE and NC molecules, decreasing the resistance of adhesive molecules to movement. At the same time, the relative flexibility of the soft segments in GAP-ETPE increases the ability of the adhesive molecule to change conformations, consequently enhancing the molecular mobility of the adhesive. The improved mobility of the adhesive molecules and the long chains of ETPE make them prone to sliding rather than tearing when subjected to impact loads. Therefore, the impact resistance of these materials was improved.

Figure 5 also shows that the ability of the three elastomers to improve the impact strength of the propellant decreases as temperatures decrease. At high temperatures, the movement space of the binder chain segments is larger and the interaction forces between the segments are weaker. The adhesive molecules have stronger mobility at 50 °C compared to −40 °C. The structural damage suffered during the deformation of the propellant under stress is often caused by the dewetting of the adhesive and RDX. This has been shown using scanning electron microscope (SEM) images of the cross-section of the R3 propellant, which can be found in Figure S2. Notably, the bonding groups of GAP-ETPE can delay the onset of dewetting, increasing the tendency of the propellant adhesive system to undergo structural damage. Consequently, CBA-ETPE, which has the strongest bond with RDX, could maximize the increase in the impact strength of the propellants. Although it is difficult to prove the existence of a delay in the dewetting described above to the direct use of SEM photos, the smaller dewetting ratio observed during the tensile fracture of the ExC propellant at 50 °C indirectly confirms this. As temperatures fall, the reduced intermolecular free volume and internal energy of the material at low temperatures restrict the molecular mobility of adhesive molecules. The increased resistance to molecular motion makes the binder undergo internal tearing more readily. This can be directly proven by the SEM image of the cross-section of the R3 propellant, as shown in Figure S3. Indeed, it is clear that the interactive forces induced by the bonding groups between the binder and RDX cannot delay the breaking of the adhesive. Consequently, it is difficult for bonding groups to generate the extra increase in the impact strength of the propellant at low temperatures.

### 3.4. Mechanical Sensitivity

**Figure 6:**
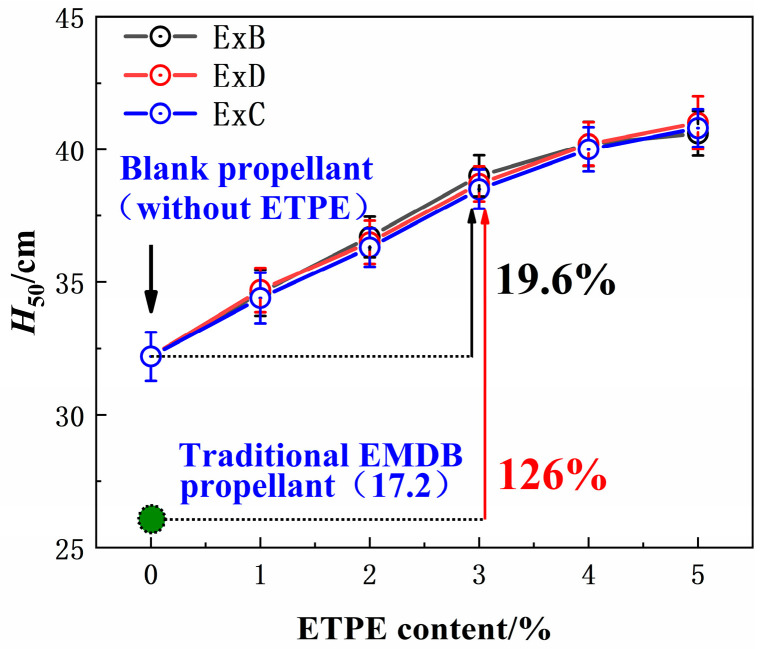
*H*_50_ of the EMDB propellant with GAP-ETPE.

Figure 6 shows the characteristic height (*H*_50_) of the EMDB propellant with GAP-ETPE. Detailed data on the sensitivity of the propellants can be found in Table S5. Figure 6 shows that the *H*_50_ of the EMDB propellant increases as the GAP-ETPE content increases. This suggests that GAP-ETPE has a positive effect on the insensitive properties of the EMDB propellant. It may be attributed to the insensitive nature and the structural characteristic of the ETPE molecule. Based on the results from the literature and the well-known hotspot theory [29,30,31], we suggest that the propellants undergo plastic deformation when subjected to mechanical stimuli, potentially forming hotspots that can lead to explosions. During the plastic deformation process, defects and voids may form within the binder system, and the closure of these voids, in a manner similar to adiabatic compression, can generate compression hotspots, potentially leading to initiation. In addition, the RDX may experience friction, shear, and compression between particles during the deformation process, leading to the formation of hotspots that can cause initiation. For EMDB propellants with high solid content, the rigid binder and the large amount of RDX can make the propellant more sensitive. The modulus of the binder system may be decreased due to GAP-ETPE, enhancing the toughness of the propellant (as evidenced by tensile test results). While this can promote the continuous plastic deformation of the binder, reducing the generation of internal defects, the reduction of its modulus means that the binder system between particles is more likely to deform and absorb external energy, reducing the rigid friction and compression between particles, allowing it to act as a buffer for the solid fillers. In other words, GAP-ETPE could partially replace RDX in its role in absorbing the energy from external stimuli, reducing the stimulation received by RDX. Together, these factors can reduce the mechanical sensitivity of the propellant.

Figure 6 also shows that the different elastomers had little effect on the *H*_50_ of the propellant. It indicates that the bonding groups in the ETPE molecule do not influence the sensitivity of the EMDB propellant. This is consistent with the aforementioned mechanical properties.

It should be noted that the R3 propellant was obtained by replacing NG in the traditional composition of the EMDB propellant with Bu-NENA, which is not sensitive to frictional stimuli. Consequently, the friction sensitivities of the three series of propellants under the influence of GAP-ETPE were all 0%. Compared to traditional EMDB propellants, the friction sensitivity of EMDB propellants with GAP-ETPE was reduced from 46% to 0%, while their *H*_50_ increased by 138% from 17.2 cm to a maximum of 41.0 cm. The friction sensitivity remained at 0% while the impact sensitivity of the propellant was further reduced when comparing the EMDB propellants to the R3 propellant. Generally, the use of GAP-ETPE results in low-sensitivity EMDB propellants.

## 4. Conclusions

In this study, a novel method of improving the interface performance between adhesives and the RDX through the incorporation of GAP-ETPE with bonding groups was proposed. The effects of molecular structure and content of ETPE on the performance of the EMDB propellants were studied. The main results and conclusions are as follows:

(1) Under the induced force between the bonding groups and the nitro groups of RDX, the interfacial compatibility between the adhesive system of the propellant and the RDX was improved, and its adhesion work increased. The cyanide group, which possesses a stronger degree of polarity, can generate stronger induced forces between the ETPE and the RDX compared to the ester groups. Consequently, the adhesive of the ExC propellant exhibited greater adhesion work with RDX compared to other propellants.
(2) All three types of ETPE improved the low-temperature elongation and impact resistance of the EMDB propellant, but BDO-ETPE and DBM-ETPE also reduced the high-temperature tensile strength of the propellant. Due to its ability to induce stronger forces, CBA-ETPE improved the mechanical properties of the propellant at low and high temperatures simultaneously. The optimal mechanical properties of the propellant were achieved at a 3% CBA-ETPE content (i.e., E3C). The elongation and impact strength of the propellant at −40 °C reached 7.49% and 6.58 MPa, respectively, while its tensile strength and impact strength at 50 °C reached 1.19 MPa and 21.1 MPa, respectively.
(3) All three types of ETPE improved the safety of the EMDB propellant. As the ETPE content increased, the sensitivity of the EMDB propellant gradually decreased. The optimal formulation for the E3C propellant had a characteristic fall height of 39.0 cm, which is 126% higher than that of the traditional EMDB propellant. In addition, the friction sensitivity of the material was reduced to 0%.

In summary, structural modification of the propellant adhesive using GAP-ETPE with bonding groups could improve the interface performance between the binder and RDX while also enhancing the mechanical properties of the propellant, including its tensile and impact strength. In other words, the novel method proposed in this work represents a simple and effective means of improving the mechanical properties of EMDB propellants, which could provide guidance for studies aiming to optimize the performance of EMDB propellants.

## Figures and Tables

**Table 1:** Abbreviations used in this work.

Abbreviation	Meaning
EMDB	Extruded modified double base
GAP	Glycidyl azide polymer
ETPE	Energetic thermoplastic elastomers
GAP-ETPE	Energetic thermoplastic elastomers based on glycidyl azide polymer
BDO	1,4-butanediol
DBM	Diethyl Bis(hydroxymethyl)malonate
CBA	N-(2-Cyanoethyl) diethanolamine
BDO-ETPE	GAP-ETPE with BDO as chain extender
DBM-ETPE	GAP-ETPE with chain extended by BDO and DBM (mass ratio 3:1)
CBA-ETPE	GAP-ETPE with chain extended by BDO and CBA (mass ratio 1:1)
R3	EMDB propellant without GAP-ETPE as a control
ExB	Propellant based on BDO-ETPE, where *x* refers to BDO-ETPE content
ExD	Propellant based on DBM-ETPE, where *x* refers to BDO-ETPE content
ExC	Propellant based on CBA-ETPE, where *x* refers to BDO-ETPE content
Ad-R3	The adhesive of R3 propellant
Ad-E3B	The adhesive of E3B propellant
Ad-E3D	The adhesive of E3D propellant
Ad-E3C	The adhesive of E3C propellant

**Table 2:** The physical properties of the three kinds of GAP-ETPE with 30% hard-segment content used in this study.

Property	BDO-ETPE	CBA-ETPE	DBM-ETPE
Average molecular weight: $\bar{M_{n}}$/g mol^−1^	30,300	29,000	30,100
Density: ρ/g cm^−3^	1.22	1.22	1.22
Glass transition temperature: *T*_g_/°C	−39.4	−38.2	−39.0

**Table 3:** Composition of EMDB propellants with different GAP-ETPE content.

Sample	NC/%	Bu-NENA/%	GAP-ETPE/%	Al/%	RDX/%	Others/%
R3	23.6	17.8	--	6	49	3.6
E1B	23.0	17.4	1	6	49	3.6
E2B	22.4	17.0	2	6	49	3.6
E3B	21.9	16.5	3	6	49	3.6
E4B	21.3	16.1	4	6	49	3.6
E5B	20.8	15.6	5	6	49	3.6
E1C	23.0	17.4	1	6	49	3.6
E2C	22.4	17.0	2	6	49	3.6
E3C	21.9	16.5	3	6	49	3.6
E4C	21.3	16.1	4	6	49	3.6
E5C	20.8	15.6	5	6	49	3.6
E1D	23.0	17.4	1	6	49	3.6
E2D	22.4	17.0	2	6	49	3.6
E3D	21.9	16.5	3	6	49	3.6
E4D	21.3	16.1	4	6	49	3.6
E5D	20.8	15.6	5	6	49	3.6

**Table 4:** Contact angles of reference liquids for different adhesive systems.

Sample	Diiodomethane/°	*N*,*N*-Dimethylformamide/°	Ethylene Glycol/°
Ad-R3	50	58	62
Ad-E3B	62	61	68
Ad-E3D	50	55	55
Ad-E3C	57	55	55

**Table 5:** The interfacial tension and adhesion work between adhesive or GAP-ETPE and RDX.

Sample	Interfacial Tension/(mJ·m^−2^)	Adhesion Work/(mJ·m^−2^)
Ad-R3	10.6	69.67
Ad-E3B	7.86	66.79
Ad-E3D	7.85	72.59
Ad-E3C	5.14	74.08

## Data Availability

Data are contained within the article.

## Supplementary Materials

The following supporting information can be downloaded at: https://www.mdpi.com/article/10.3390/polym16060792/s1, Figure S1: The synthesis process diagram of three kinds GAP-ETPE: (a) BDO-ETPE; (b) DBM-ETPE; (c) CBA-ETPE; Table S1: The tensile properties of EMDB propellants at 50 °C with GAP-ETPE; Table S2: The tensile properties of EMDB propellants at 20 °C with GAP-ETPE; Table S3: The tensile properties of EMDB propellants at −40 °C with GAP-ETPE; Table S4: The impact strength of EMDB propellants with GAP-ETPE; Table S5: The mechanical sensitivity of EMDB propellants with GAP-ETPE; Figure S2: SEM image of cross section R3 propellant at 50 °C; Figure S3: SEM image of cross section R3 propellant at −40 °C.
