# Supplementary material for: A Novel Method of Improving the Mechanical Properties of Propellant Using Energetic Thermoplastic Elastomers with Bonding Groups
Source: Polymers (Basel). 2024 Mar 13;16(6):792. doi: 10.3390/polym16060792 (PMC10975028; doi:10.3390/polym16060792)
Supplement: Supplementary file 1 [file polymers-16-00792-s001.zip › polymers-2907268-supplementary.pdf]

Supporting Information

A novel method of improving the mechanical properties of propellant using energetic thermoplastic elastomers with bonding groups

The Authors:

Shixiong Sun [1,2], Haoyu Liu [1], Yang Wang[1], Wenhao Du [1,2], Benbo Zhao [1, 2,*] and Yunjun Luo [3,*]

Corresponding Author: Prof. Benbo Zhao; Prof. Yunjun Luo

Address:

1 School of Chemical Engineering and Technology, North University of China, Taiyuan 030051, China

2 Dezhou Industrial Technology Research Institute of North University of China, Dezhou 253034, China

3 School of Materials Science and Engineering, Beijing Institute of Technology, 5 South Zhongguancun Street, Beijing 100081, China.

Tel.: +86-10-3922315; +86-10-68913698

E-mail: zhaobenbo@163.com (B. Zhao); yjluo@bit.edu.cn (Y. Luo).

The synthesis process of three kinds GAP-ETPE are as follows:

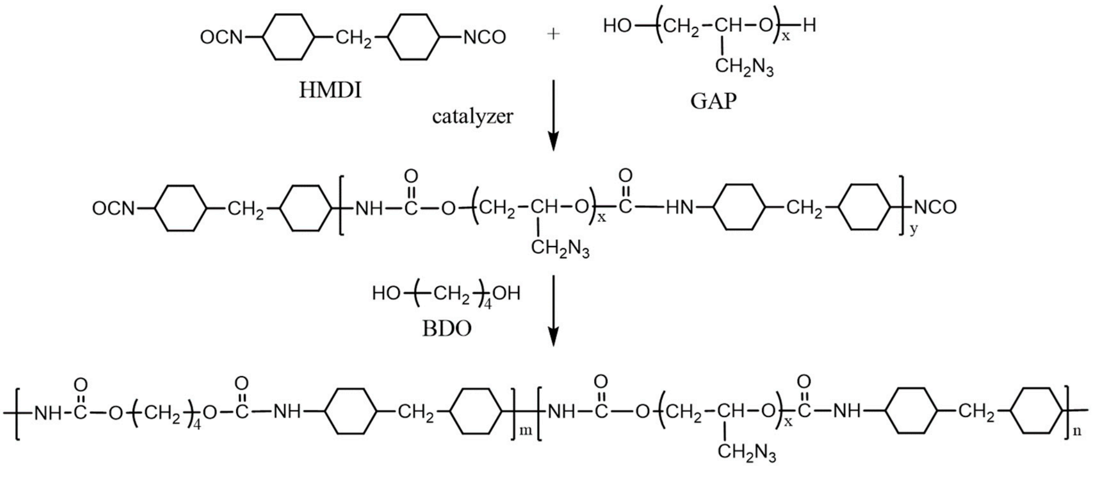

(a) BDO-ETPE

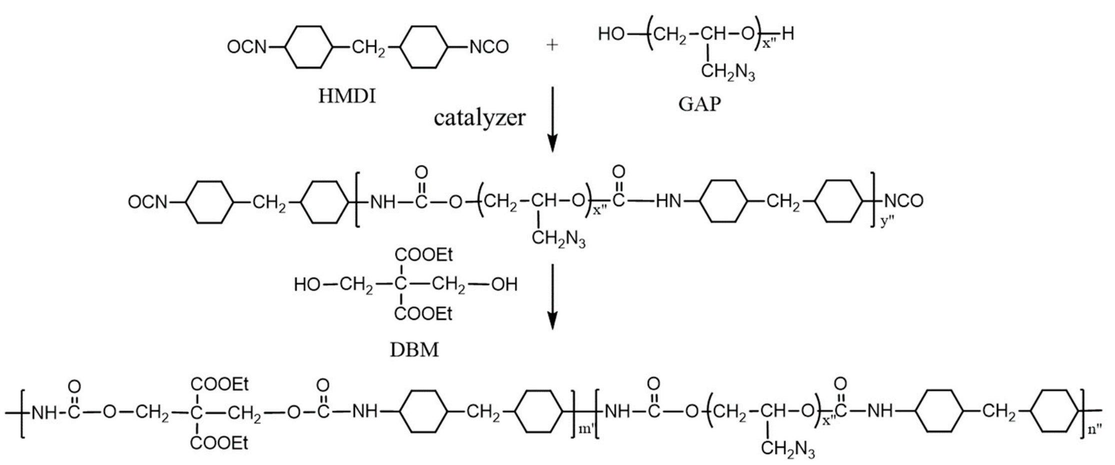

(b) DBM-ETPE

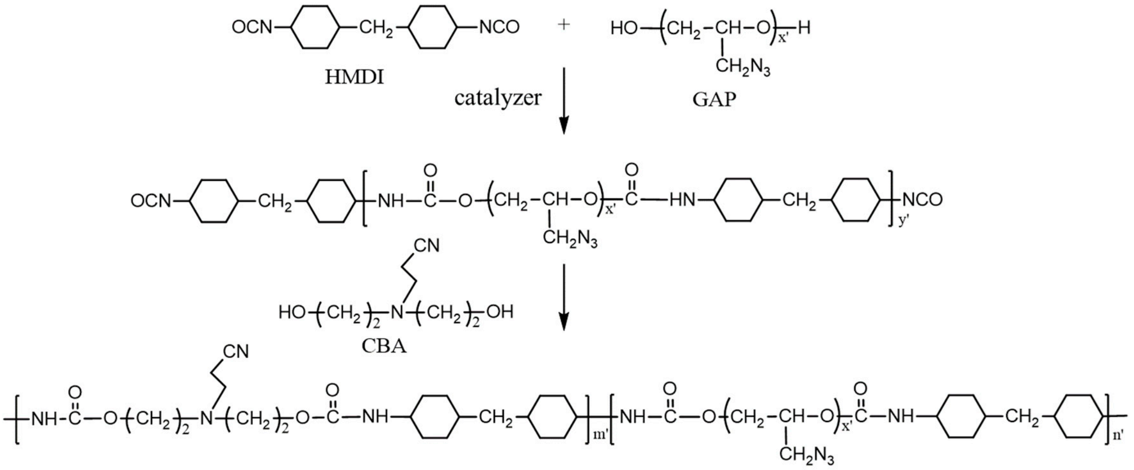

(c) CBA-ETPE

Figure S1. The synthesis process diagram of three kinds GAP-ETPE: (a) BDO-ETPE; (b) DBM-ETPE; (c) CBA-ETPE.

Table S1. The tensile properties of EMDB propellants at 50 °C with GAP-ETPE.

Sample	σ_m/MPa	ε_m/%	σ_b/MPa	ε_b/%	$\varepsilon_b/\varepsilon_m$	E/MPa
R3	1.11	18.86	0.96	21.74	1.16	14.71
E1B	1.09	21.03	1.00	23.55	1.12	12.96
E2B	1.02	22.54	0.98	26.60	1.18	11.31
E3B	0.99	25.34	0.98	29.65	1.17	9.77
E4B	0.88	26.21	0.85	30.46	1.20	8.39
E5B	0.83	25.58	0.79	31.21	1.22	8.11
E1D	1.10	20.93	0.89	23.02	1.10	13.14
E2D	1.10	22.59	0.95	24.93	1.10	12.17

E3D	1.04	23.13	0.82	25.54	1.10	11.24
E4D	0.90	24.90	0.76	27.10	1.08	9.04
E5D	0.85	26.01	0.73	28.09	1.08	8.17
E1C	1.10	20.68	1.06	22.65	1.09	13.30
E2C	1.17	21.21	1.04	23.47	1.10	13.79
E3C	1.19	22.08	0.94	23.75	1.08	13.47
E4C	1.04	22.50	0.88	24.28	1.08	11.56
E5C	0.95	24.39	0.84	26.58	1.09	9.74

Note: σ_m is the maximum tensile strength; ε_m is the elongation at maximum tensile strength; σ_b is the fracture strength; ε_b is the fracture elongation. E is the tensile modulus.

Table S2. The tensile properties of EMDB propellants at 20 °C with GAP-ETPE.

Sample	σ_m/MPa	ε_m/%	σ_b/MPa	ε_b/%	$\varepsilon_b/\varepsilon_m$	E/MPa
R3	5.41	9.06	3.62	9.96	1.10	131.3
E1B	4.86	10.41	3.18	11.48	1.10	102.7
E2B	4.65	10.61	3.13	11.79	1.11	96.4
E3B	4.22	12.52	3.23	13.83	1.10	74.1
E4B	4.09	15.96	3.75	17.39	1.09	56.3
E5B	3.69	18.56	2.97	20.12	1.08	43.7
E1D	5.14	9.45	4.03	10.27	1.09	119.6
E2D	4.99	9.99	3.43	10.95	1.10	109.8
E3D	4.67	10.19	3.99	11.27	1.10	100.8
E4D	4.36	11.78	3.17	13.08	1.11	81.43
E5D	4.28	12.88	3.35	14.28	1.11	73.11
E1C	5.33	8.82	4.42	9.91	1.12	132.9
E2C	5.13	9.70	3.97	10.20	1.05	116.3
E3C	4.97	10.14	3.14	10.85	1.07	107.8
E4C	4.84	11.79	3.28	12.88	1.09	90.31
E5C	4.66	12.62	3.36	13.86	1.10	81.24

Table S3. The tensile properties of EMDB propellants at −40 °C with GAP-ETPE.

Sample	σ_m/MPa	ε_m/%	σ_b/MPa	ε_b/%	$\varepsilon_b/\varepsilon_m$	E/MPa
R3	24.98	7.08	24.98	7.09	1.00	740.93
E1B	24.66	7.10	24.66	7.10	1.00	729.38
E2B	24.38	7.27	24.38	7.27	1.00	704.24
E3B	24.03	7.42	24.03	7.42	1.00	680.09
E4B	23.81	7.68	23.81	7.68	1.00	651.05
E5B	23.23	7.74	23.23	7.74	1.00	630.27
E1D	24.59	7.08	24.59	7.08	1.00	729.36

E2D	24.24	7.21	24.24	7.21	1.00	706.02
E3D	23.80	7.45	23.80	7.45	1.00	670.87
E4D	23.50	7.58	23.50	7.58	1.00	651.06
E5D	23.08	7.79	23.08	7.79	1.00	622.18
E1C	24.59	7.13	24.59	7.13	1.00	724.25
E2C	23.97	7.28	23.97	7.28	1.00	691.44
E3C	23.81	7.49	23.81	7.49	1.00	667.57
E4C	23.50	7.66	23.50	7.66	1.00	644.26
E5C	22.93	7.73	22.93	7.73	1.00	622.94

Table S4. The impact strength of EMDB propellants with GAP-ETPE.

Sample	Impact strength/$kJ \cdot m^{-2}$		
	−40 °C	20 °C	50 °C
Traditional EMDB	3.54	6.98	10.22
R3	5.50	9.14	16.38
E1B	5.71	9.88	16.65
E2B	5.98	10.54	17.14
E3B	6.48	12.22	18.17
E4B	6.79	13.07	19.08
E5B	7.17	13.84	-
E1D	5.46	10.03	17.46
E2D	5.94	11.13	18.22
E3D	6.59	12.41	18.92
E4D	6.95	13.75	21.16
E5D	7.33	14.23	-
E1C	5.59	10.44	16.68
E2C	6.00	10.85	19.15
E3C	6.58	12.57	21.14
E4C	6.88	13.61	22.30
E5C	7.21	14.32	-

Table S5. The mechanical sensitivity of EMDB propellants with GAP-ETPE.

Sample	H_{50}/cm	P/%
Traditional EMDB	17.2	46
R3	32.2	0
E1B	34.6	0
E2B	36.7	0
E3B	39.0	0
E4B	40.2	0
E5B	40.6	0

E1C	34.4	0
E2C	36.3	0
E3C	38.5	0
E4C	40.0	0
E5C	40.8	0
E1D	34.7	0
E2D	36.5	0
E3D	38.7	0
E4D	40.2	0
E5D	41.0	0

The interfacial tension and adhesion work between Bu-NENA/NC/GAP-ETPE adhesive system and RDX were calculated due the following formula. Specifically, the interfacial tension between two phases can be expressed as

$$\gamma_{\mathrm{sl}} = \gamma_{\mathrm{s}} - \gamma_{\mathrm{l}} \cos\theta \qquad (\theta > 0) \tag{1}$$

where θ is the contact angle, γ_{s} is the solid surface tension, γ_{l} is the liquid surface tension and γ_{sl} is the interfacial tension between the two. In addition, the surface energy (γ) was made up of a dispersion component (d) and a polarity component (p), and γ_{sl} can be expressed as the following equation:

$$\gamma_{\mathrm{sl}} = \gamma_{\mathrm{s}} + \gamma_{\mathrm{l}} - 2\sqrt{\gamma_{\mathrm{s}}^{\mathrm{d}}}\sqrt{\gamma_{\mathrm{l}}^{\mathrm{d}}} - 2\sqrt{\gamma_{\mathrm{s}}^{\mathrm{p}}}\sqrt{\gamma_{\mathrm{l}}^{\mathrm{p}}}$$

$$= \left(\gamma_{\mathrm{s}}^{\mathrm{d}} + \gamma_{\mathrm{s}}^{\mathrm{p}}\right) + \left(\gamma_{\mathrm{l}}^{\mathrm{d}} + \gamma_{\mathrm{l}}^{\mathrm{p}}\right) - 2\sqrt{\gamma_{\mathrm{s}}^{\mathrm{d}}}\sqrt{\gamma_{\mathrm{l}}^{\mathrm{d}}} - 2\sqrt{\gamma_{\mathrm{s}}^{\mathrm{p}}}\sqrt{\gamma_{\mathrm{l}}^{\mathrm{p}}} \tag{2}$$

where γ_s^d and γ_s^p are the dispersion and polarity components of the solid, respectively, and γ_l^d and γ_l^p are the dispersion and polarity components of the liquid separately.

Based on Eqns (1) and (2), a new formula can be derived

$$\gamma_{\mathrm{l}}(1 + \cos\theta)/2\sqrt{\gamma_{\mathrm{l}}^{\mathrm{d}}} = \sqrt{\gamma_{\mathrm{s}}^{\mathrm{d}}} + \sqrt{\gamma_{\mathrm{s}}^{\mathrm{p}}} \times \sqrt{\gamma_{\mathrm{l}}^{\mathrm{p}}}/\sqrt{\gamma_{\mathrm{l}}^{\mathrm{d}}} \tag{3}$$

Then the values of γ_s^d, γ_s^p and γ_s can be obtained. The interfacial tension (γ_{s1s2}) and work of adhesion (W_{a}) between the binders and explosives can be calculated using the following equations:

$$\gamma_{s1s2} = \gamma_{s1} + \gamma_{s2} - 2\sqrt{\gamma_{s1}^{d}}\sqrt{\gamma_{s2}^{d}} - 2\sqrt{\gamma_{s1}^{p}}\sqrt{\gamma_{s2}^{p}} \quad (4)$$

$$W_a = \gamma_{s1} + \gamma_{s2} - \gamma_{s1s2} \quad (5)$$

where γ_{s1} and γ_{s2} represent the surface tensions of solid 1 (s1) and solid 2 (s2) separately, γ_{s1}^{d} and γ_{s2}^{d} denote the dispersion component of s1 and s2 separately, γ_{s1}^{p} and γ_{s2}^{p} are the polarity component of s1 and s2 separately, and the γ_{s2} of RDX used here was theoretical values.

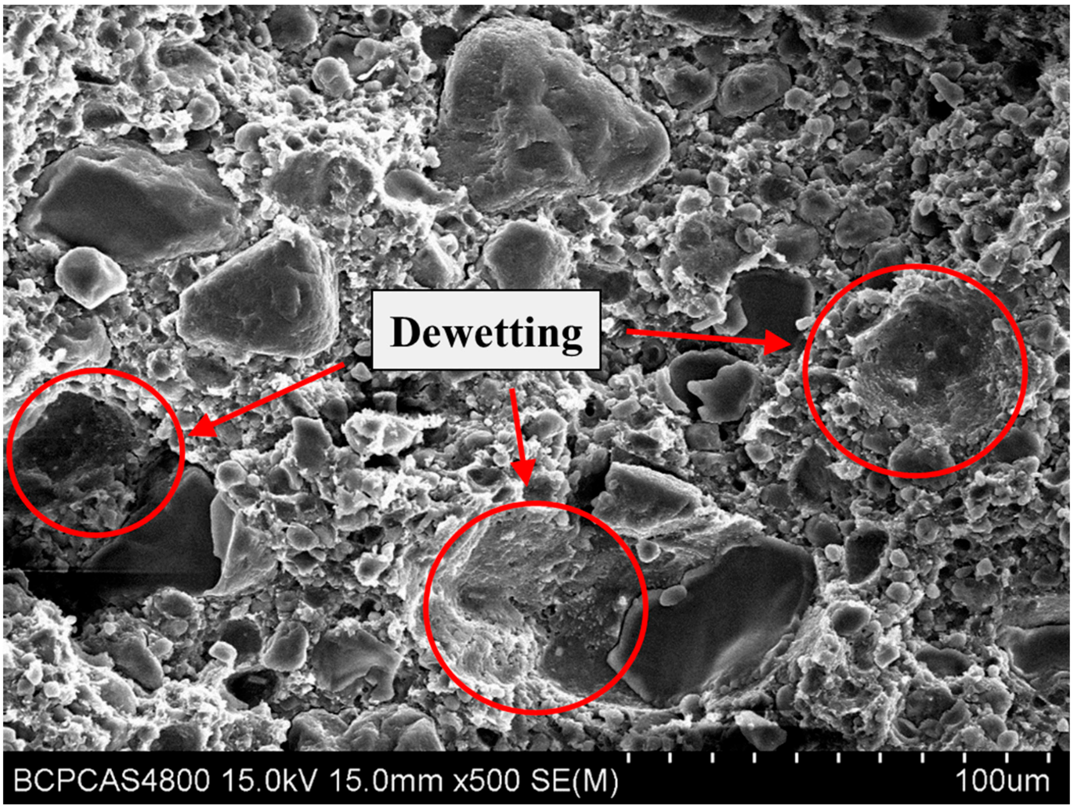

Figure S2. SEM image of cross section R3 propellant at 50°C

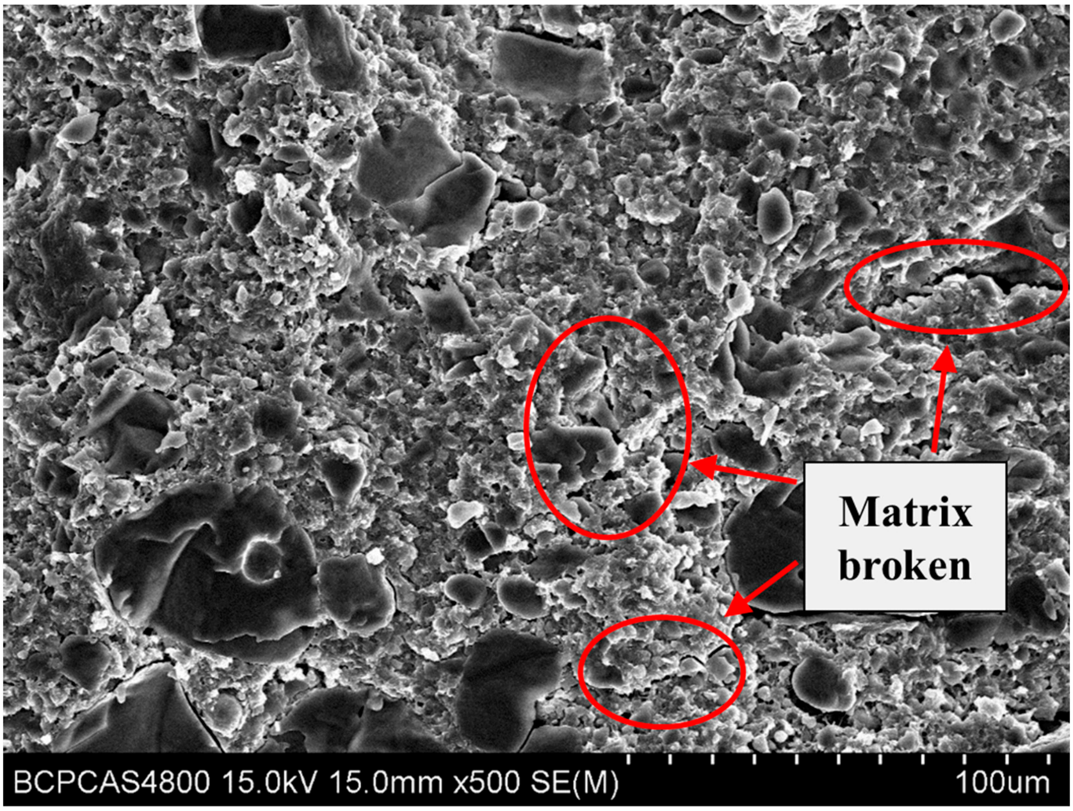

Figure S3. SEM image of cross section R3 propellant at −40℃
